# Mitigating Stress‐Induced Nonphotoactive Phase Transition Through Sodium Sulfonate Engineering for Stable and Efficient Perovskite Solar Cells

**DOI:** 10.1002/anie.2425823

**Published:** 2026-08-05

**Authors:** Zhihuan Tang, Mingzi Sun, Jinghao Ge, Ying Wang, Yunfan Wang, Yiran Tao, Lu Zhang, Xinhui Lu, Sai‐Wing Tsang, Weizhong Tian, Rui Wang, Hao‐Chung Kuo, Bolong Huang, Shengzhong (Frank) Liu, Jiaxue You, Alex K. Y. Jen

**Affiliations:** ^1^ Institute For Advanced Energy Materials School of Materials Science and Engineering Shaanxi Normal University Xi'an China; ^2^ Department of Chemistry City University of Hong Kong Kowloon Hong Kong SAR China; ^3^ Department of Materials Science and Engineering Hong Kong Institute for Clean Energy City University of Hong Kong Kowloon Tong Hong Kong SAR China; ^4^ Department of Physics The Chinese University of Hong Kong Shatin Hong Kong SAR China; ^5^ Department of Materials Science and Engineering School of Engineering Zhejiang Provincial Key Laboratory of Intelligent Low‐Carbon Biosynthesis Westlake University Hangzhou China; ^6^ Semiconductor Research Center Hon Hai Research Institute Taipei Taiwan China; ^7^ Key Laboratory of Photoelectric Conversion and Utilization of Solar Energy Dalian Institute of Chemical Physics Chinese Academy of Sciences Dalian Liaoning China; ^8^ Center of Materials Science and Optoelectronics Engineering University of Chinese Academy of Sciences Beijing China

**Keywords:** crystallization kinetics, high efficiency, lattice geometry, perovskite solar cells, thermal and light stability

## Abstract

Formamidinium lead triiodide (FAPbI_3_) perovskite solar cells (PSCs) have attracted significant attention due to their outstanding optoelectronic properties. However, their long‐term stability remains limited by lattice strain‐induced transition from the photoactive α‐phase to the nonphotoactive δ‐phase. In this work, first‐principles calculations reveal that the incorporation of Na^+^ into interstitial sites between adjacent FA^+^ cations significantly reduces the formation energy of the α‐phase, thereby promoting its thermodynamic stabilization. Then, experimental results confirm that the introduction of 2 mol% Na^+^ effectively alleviates lattice strain while simultaneously suppressing δ phase. Moreover, the accompanying sulfonate groups interacting with PbI_2_ can regulate the crystallization and improve film quality. As a result, the optimized PSC achieved power conversion efficiency (PCE) as high as 26.67% (certificated 26.44%), ranking among the highest reported for the n‐i‐p structured devices. Notably, the bare device without encapsulation retained over 90% of its initial efficiency after continuous heating at 85°C for 1200 h and maintained 80% after 800 h continuous illumination. This study demonstrates that metal cation doping is an effective strategy for stabilizing the perovskite lattice and enhancing long‐term operational stability of perovskite‐based optoelectronic devices.

## Graphical abstract

To address the phase stability issue of α‐FAPbI_3_, we employed a cation doping strategy using 1‐decanesulfonate (C_10_H_21_NaO_3_S). This doping releases lattice strain and suppresses the formation of the δ‐phase, enabling breakthrough performance in perovskite solar cells with a power conversion efficiency of 26.67% and excellent thermal and photostability.

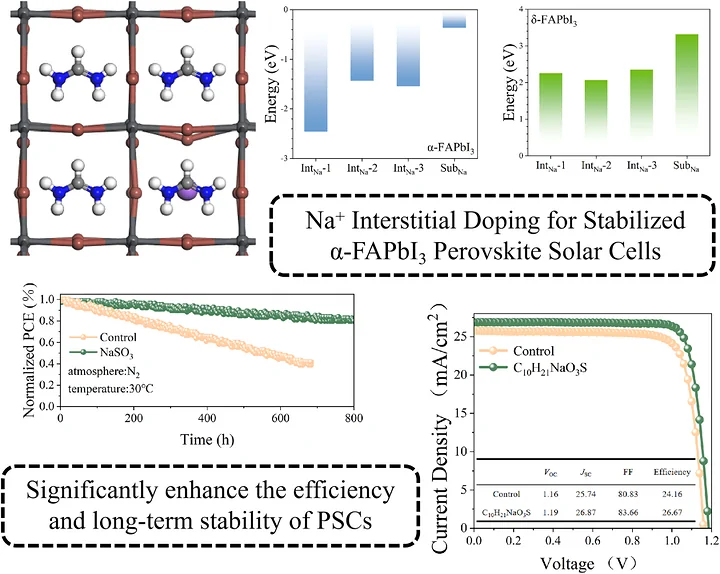

## 1 Introduction

In recent years, perovskite solar cells (PSCs) have garnered considerable attention as promising next‐generation photovoltaic devices owing to their remarkable properties [1]. So far, the power conversion efficiency (PCE) of PSCs has reached over 27% [2, 3, 4, 5, 6, 7, 8, 9]. The structure of lead halide perovskite is represented by the general formula ABX_3_, where A represents organic ammonium or inorganic cation, B metal cation, and X halides [10]. At present, high‐efficiency PSCs are mainly manufactured using FAPbI_3_ [11]. However, due to lattice expansion triggered by the FA^+^ cation, the stability of α‐FAPbI_3_ is diminished, leading to a spontaneous degradation into the nonphotoactive yellow δ‐phase [12]. One of the most common strain compensation strategies is to simultaneously introduce smaller ions to reduce local strains in the perovskite lattice [13].

The strain in the FAPbI_3_ perovskite can be reduced by replacing some FA^+^ ions with small‐sized cations [14]. Zhu et al. [15] found that reducing the effective tolerance factor of FAPbI_3_ by adding the smaller‐size Cs^+^ cation can stabilize the α‐phase perovskite structure. He et al., [16] observed that Rb^+^ ions can fill the cation vacancies caused by MA volatilization during the annealing stage, reducing the shrinkage strain of perovskite crystals. Park at al [17] observed that partially replacing FA^+^ with MA^+^ in FAPbI_3_ can enhance the stability of the α‐phase of FAPbI_3_. Cs^+^ is the common cation used to regulate lattice strain in FAPbI_3_ perovskite [18]. Also, K^+^ and Rb^+^ were used to adjust the lattice strain in FAPbI_3_ perovskite [19]. Guo at al [20] observed that low concentration doping of K^+^ in CsPbBr_3_ can replace some A‐site cations, leading to a decrease in lattice strain of perovskite. The radii of K^+^, Rb^+^, and Cs^+^ are 138, 152, and 167 pm, respectively [21]. The radius of FA^+^ is around 253 pm and MA^+^ is around 217 pm [22]. Goldschmidt tolerance factor (*t*) is an empirical index for predicting stable crystal structures of perovskite materials. Equation (1) is the calculation formula for the Goldschmidt tolerance factor (*t*):

(1) $$t=\frac{R_{A}+R_{B}}{\sqrt{2}\left(R_{B}+R_{X}\right)}\;$$

where *t* is the Goldschmidt tolerance factor, *R*_A_ is the radius of A cation, *R*_B_ is the radius of B cation and *R*_X_ is the radius of the anion. In inorganic–organic hybrid perovskites, the organic cation usually has a nonspherical geometry and rotates constantly in the lattice [23, 24]. A *t* value between 0.8 and 1.1 is favorable for cubic perovskite structure, and larger (>1.1) or smaller (<0.8) values of tolerance factor usually result in nonperovskite structures [25]. The alloying compositions have smaller tolerance factors than pure FAPbI_3_. The effective tolerance factor is reduced to relax local lattice strain, and to improve both efficiency and stability in solar cells [12].

On the other hand, FAPbI_3_ undergoes a complex phase transition from its precursor solution to the solid‐phase alpha (α) phase. In this phase transition process, the nucleation control is crucial for the final film quality. Ge et al. [26]. demonstrated that aminoguanidine hydrochloride could form an intermediate phase and modulate nucleation in the FAPbI_3_ system. Satoshi Uchida et al. [27] investigated the effect of chloride integration on the nucleation of perovskite films. Therefore, crystallization optimization is also crucial for preparing high‐quality PSCs. Tao et al. [28] introduced 2‐aniline‐4‐sulfonic acid containing sulfonic acid groups into the perovskite system, delayed the crystallization kinetics, and prepared high crystallinity, large grain perovskite films.

In this study, we employed first‐principles calculations to investigate whether sodium ions (Na^+^) can incorporate into the formamidinium (FA) perovskite lattice to stabilize the α‐phase. The calculations have revealed that Na^+^ doping significantly impacts the phase stability and electronic structures of FAPbI_3_ perovskite with distinct preferences for doping sites and energy characteristics. Interstitial sites are more favored than substitutional sites for α‐FAPbI_3_ during the Na^+^ doping. Notably, α‐FAPbI_3_ exhibits much lower formation energies of Na^+^ doping than δ‐FAPbI_3_ across all possible doping sites, enabling the stabilization of the α phase over the δ phase. The sulfonic acid ions (C_10_H_21_O_3_S^−^) were used to balance the charge of Na^+^ cations (Figure S1) and interacted with PbI_2_ to regulate the crystallization process, effectively suppressing the precipitation of PbI_2_. Finally, optimized PSCs achieved a champion efficiency of 26.67%. In addition, the optimized devices exhibit excellent thermal stability and light stability, with an initial efficiency of over 90% after storage at 85°C for 1200 h. And after 800 h of illumination in an N_2_ atmosphere, it maintained at 80% of the initial efficiency.

## 2 Results and Discussion

### 2.1 First‐Principles Calculations Design

To investigate the influences of Na^+^ doping on the perovskite lattice, we have performed theoretical calculations based on the FAPbI_3_ lattice. After introducing the Na^+^ doping, we have considered three different interstitial sites and one substitutional site for both α‐FAPbI_3_ and δ‐FAPbI_3_ (Figure 1a–d, Figure S3). Int_Na_‐1 is the interstitial site between FA molecules arranged in parallel with Na^+^ in α‐FAPbI_3_ (Figure 1a). Int_Na_‐2 is located in the interstitial site of the Pb‐I octahedral inorganic framework, near the position of the I atom (Figure 1b). Int_Na_‐3 is an interstitial site formed by the aggregation of multiple FA molecules, with Na^+^ located near the edge of the Pb‐I octahedral skeleton (Figure 1c). Sub_Na_ is a substitutional doping, where Na^+^ ions directly replace the lattice sites of Pb^2+^ in the α‐FAPbI_3_ crystal, rather than occupying interstitial positions (Figure 1d). Transmission electron microscopy (TEM) analysis reveals that the lattice spacing decreased from 3.28to 3.15 Å after doping with Na^+^, providing evidence for the incorporation of Na^+^ within the perovskite crystal lattice (Figure S4). According to Bragg's law, the shift of the diffraction peak to higher angles (Figure 2b) is caused by a decrease in the interplanar crystal spacing. The decrease in lattice spacing indicates the introduction of some compressive stress. Lan et al. [29]. indicated that the introduced compressive stress, attributed to sodium interstitial doping, clamps the perovskite lattice from all directions, thereby raising the activation barrier for the transition from α‐to‐δ phase.

**FIGURE 1:**
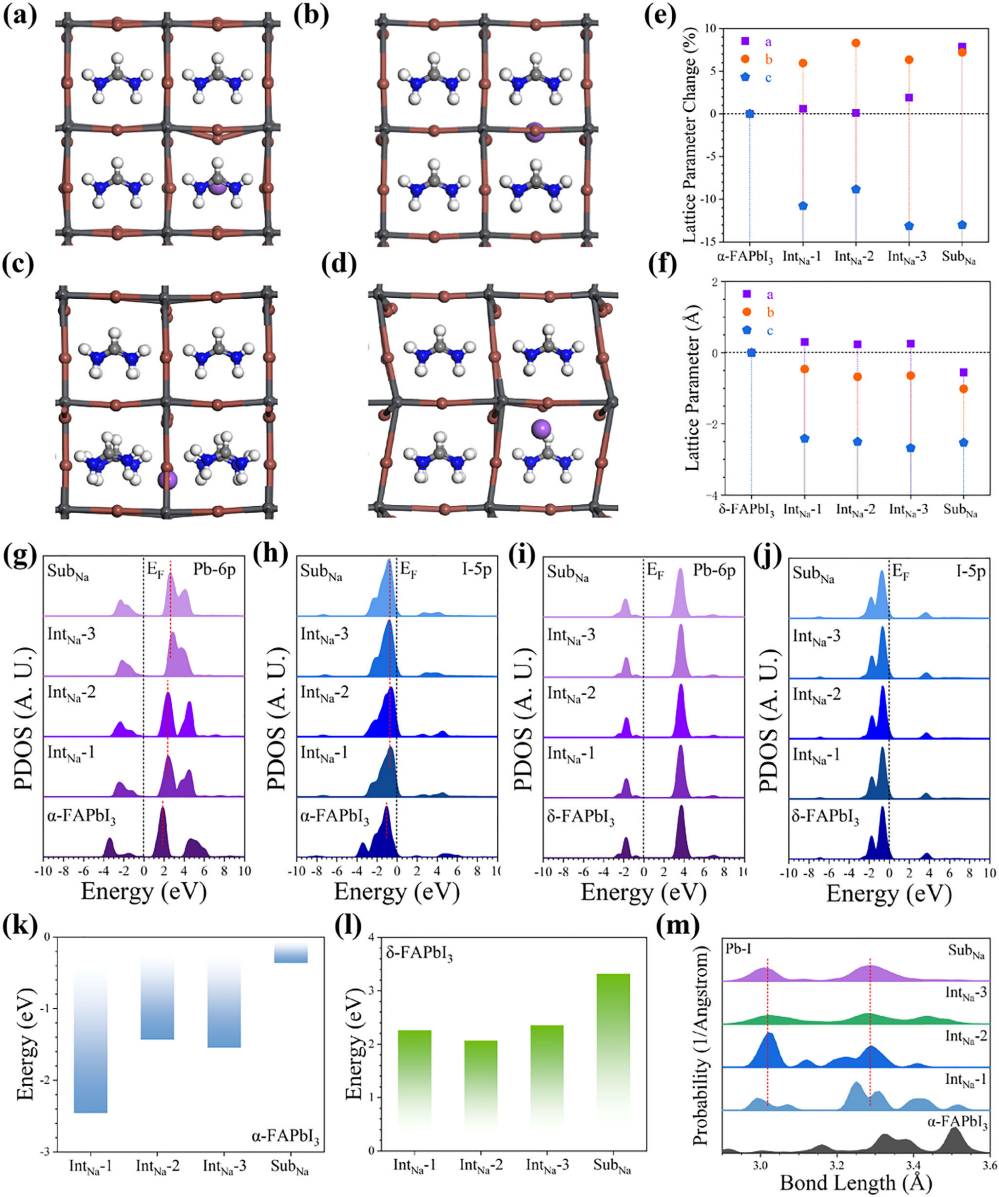
The relaxed structures of the α‐FAPbI_3_ with different doping sites. (a) IntNa‐1, (b) IntNa‐2, (c) IntNa‐3, and (d) SubNa. Dark grey balls = Pb, brown balls = I, purple balls = Na, grey balls = C, blue balls = N, and white balls = H. The lattice parameter changes in (e) α‐FAPbI_3_ and (f) δ‐FAPbI_3_. a, b, and c refer to the unit cell axes. The comparisons of (g) Pb‐6p and (h) I‐5p in the α‐FAPbI_3_. The comparisons of (i) Pb‐6p and (j) I‐5p in the δ‐FAPbI_3_. The formation energy of Na^+^ doping in (k) α‐FAPbI_3_ and (l) δ‐FAPbI_3_. The bond length changes of Pb‐I induced by Na^+^ doping in the α‐FAPbI_3_.

**FIGURE 2:**
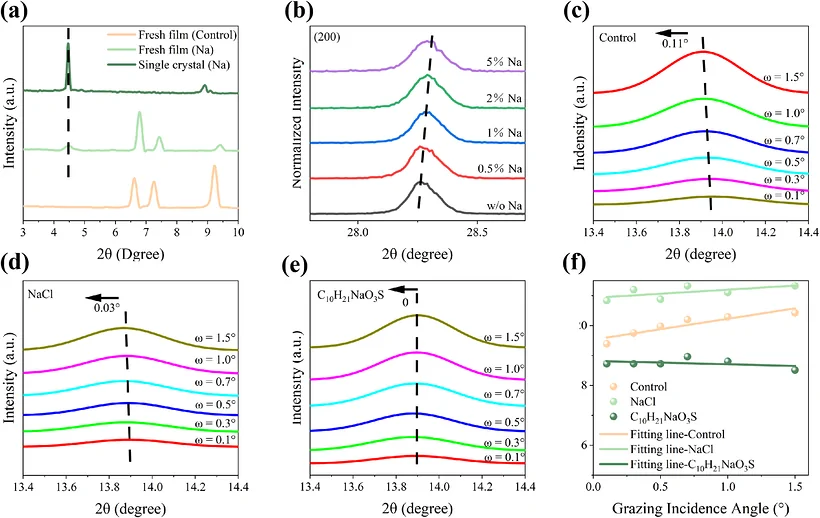
Lattice geometry optimization in perovskites. (a) XRD reflections of fresh perovskite films and single crystal. (b) XRD reflections of the (200) plane of perovskite films containing different amounts of Na^+^. GIXRD spectra of (c) the Control perovskite film, (d) the NaCl‐modified perovskite film and (e) the C_10_H_21_NaO_3_S‐modified perovskite film measured at different grazing‐incidence angles. (f) The d‐spacing value of the (100) plane as a function of grazing‐incidence angle for the Control, NaCl‐modified and C_10_H_21_NaO_3_S‐modified perovskite films.

The lattice parameters after full geometry relaxations have shown that the α‐FAPbI_3_ is more sensitive to the Na^+^ doping, where a stronger structural distortion is noted, especially the shrinkage in the c‐axis (Figure 1e). Among different sites, the interstitial Na^+^ ions between the aligned FA^+^ (IntNa‐1 and IntNa‐2) display the smallest influence on the lattice structure with the lowest distortion level. The lattice parameter c exhibits the most evident decrease when compared to other axes a and b, leading to the reduced volume after Na^+^ doping. For the δ‐FAPbI_3_, the overall lattice volume also displays a shrinkage trend, which also reveals the evident decreasing trend of the *c*‐axis (Figure 1f). Compared to the α‐FAPbI_3_, the overall distortion level in the δ‐FAPbI_3_ is smaller due to the much larger void space in the δ‐FAPbI_3_. Different from the α‐FAPbI_3_, the substitution of Na^+^ in the δ‐FAPbI_3_ induces a smaller distortion than the interstitial doping. With the lattice structures, we have also compared their electronic structure changes. For the α‐FAPbI_3_, both Pb‐6p orbitals and I‐5p orbitals show the upshifting trend, where the stronger distortions result in more shifting of the p orbitals (Figure 1 g,h). In comparison, the doping of Na^+^ shows very limited influences on the electronic structures of δ‐FAPbI_3_, where the Pb‐6p and I‐5p orbitals remain unchanged (Figure 1i,j).

Although the δ‐FAPbI_3_ displays higher structural tolerance to the Na^+^ doping, the introduction of Na^+^ doping meets much higher energy costs. Apparently, the formation energies of Na^+^ doped α‐FAPbI_3_ are much lower than those of δ‐FAPbI_3_ in all different sites, supporting that the Na^+^ doping facilitates the stabilization of the α phase of perovskite (Figure 1k,l). The interstitial Na^+^ doping is more preferred than the substitutional Na^+^ doping for both α‐FAPbI_3_ and δ‐FAPbI_3_. Among the three possible interstitial sites in the α‐FAPbI_3_, IntNa‐1 shows the lowest formation energy as the most favored doping site. For the δ‐FAPbI_3_, the energy costs for interstitial doping are similar. In addition, the Pb─I bond length distributions are compared with those after Na^+^ doping in the α‐FAPbI_3_ (Figure 1m). Notably, the overall bond length distributions for pristine α‐FAPbI_3_ are mainly located in ∼3.3 and ∼3.5 Å, which downshift to ∼3.0 and ∼3.3 Å, respectively, after the introduction of Na^+^ doping, accounting for the overall volume changes. To further evaluate the influences of Na^+^ doping, we compared the transition barriers for the α‐to‐δ phase conversion with and without Na^+^ doping (Figure S5). Although the δ‐FAPbI_3_ remains thermodynamically more stable than the α phase with a lower free energy of −0.31 eV, the Na^+^ doping substantially increases the kinetic barrier from 0.63 to 1.06 eV for the α‐to‐δ phase transformation. This indicates that Na^+^ doping effectively suppresses the spontaneous α‐to‐δ conversion via kinetic inhibition of the phase transition.

To confirm that Na^+^ doping can suppress the phase transition from α‐FAPbI_3_ to δ‐FAPbI_3_, we subjected the pristine control perovskite film and Na^+^‐doped perovskite film to continuous one‐sun illumination for 158 h (Figure S6). After the illumination test, a yellow phase (δ‐FAPbI_3_) was clearly observed on the pristine control film, whereas the Na^+^‐doped perovskite film remained in the black α‐FAPbI_3_ phase without obvious phase degradation.

### 2.2 Stress Regulation

We performed energy‐dispersive x‐ray spectroscopy (EDS) analysis on perovskite using transmission electron microscopy (TEM) to display the distribution of Na and S elements (Figure S7). We observed that Na and S elements were uniformly distributed in the perovskite. We conducted a full x‐ray photoelectron spectroscopy (XPS) characterization (Figure S8). It confirmed the existence of Na element over FAPbI_3_ with a Na 1s peak (1071 eV), which is consistent with the previously reported. According to DFT calculations, the interstitial sites are preferred over substitutional ones for Na^+^ doping. We conducted x‐ray diffraction (XRD) measurements of fresh film and single crystals (Figure 2a). Compared to the control perovskite film, the Na^+^‐doped perovskite film and single crystal exhibit new peaks at 4.5°. And we conducted XRD measurements of perovskite films with different concentrations of Na^+^ (0, 0.5, 1, 2, and 5 mol%) (Figure 2b). The addition of Na^+^ ions caused a shift in the XRD peaks toward higher angles. The shift of diffraction peaks toward higher angles indicates lattice contraction, resulting compressive stress which can effectively stabilize α‐FAPbI_3_ [30, 31].The same result is observed for the (100) crystal plane (Figure S9). According to Bragg's law, the shift of the diffraction peak to lower angles is caused by an increase in the interplanar crystal spacing, resulting in lattice expansion, which is manifested as pressure and strain [32]. When the peak position is converted into the lattice constant, we can infer an isotropic decrease in the unit cell size by using Equation (2).

(2) $$a=\frac{d_{\mathrm{hkl}}}{\sqrt{h^{2}+k^{2}+l^{2}}},$$

where *a* is the lattice constant, *d*_hkl_ is the interplanar spacing of the (hkl) plane, and h, k, and l are the Miller indices.

To investigate the lattice strain distribution in the depth direction of the perovskite film, we selected the (100) crystal plane for grazing incidence x‐ray diffraction (GIXRD) analysis with variable incident angles (Figure 2c–e). When the incident angle (*ω*) increases from 0.1° to 1.5°, the diffraction peak of the Control perovskite film at 2*θ* = 13.95° gradually shifts to lower angles. And the diffraction peak of the NaCl‐modified perovskite film shifts less, which is attributed to the contraction of the *c*‐axis induced by Na^+^ doping, accompanied by the introduction of appropriate compressive strain. Besides, the diffraction peak of the C_10_H_21_NaO_3_S‐modified perovskite film shows almost no change, regardless of *ω*. This is because the sulfonate group participates in regulating the crystallization process, thereby promoting stress relaxation. Moreover, when the *ω* increased from 0.1° to 1.5°, a significant increase in the d‐spacing of the Control perovskite film was observed. And the d‐spacing of the NaCl‐modified perovskite film changed less than that of the Control perovskite film. Besides, the d‐spacing of the C_10_H_21_NaO_3_S‐modified perovskite film hardly changed (Figure 2f). This indicates that the residual stress in the perovskite film is almost completely relieved after the addition of Na^+^.

### 2.3 Crystallization Optimization

To understand which component of the compound (Na^+^ or C_10_H_21_O_3_S^−^) is important for improving the film quality during crystallization, we compare the Control perovskite film, NaCl‐modified perovskite film and C_10_H_21_NaO_3_S‐modified perovskite film. To gain insights into the remarkably enhanced film quality, we adopted in situ photoluminescence (PL) spectrometry to understand the role of Na^+^ and C_10_H_21_O_3_S^−^ during crystallization. In the initial stage of spin coating (∼0‐5 s), the precursor wet film did not form an ordered crystal phase due to the solvation effect, so no obvious PL signal was detected. When an antisolvent is introduced, the solution quickly reaches a supersaturated state, triggering nucleation [33, 34], at which point the initial PL emission of nanocrystalline nuclei can be observed (Figure S10). As the crystallization continues, a systematic red shift of the PL peak is observed [35, 36]. In the Control perovskite film, it shifts from 704.14 to 765.71 nm; in the NaCl‐modified perovskite film, from 707.16 to 770.94 nm; but in the C_10_H_21_NaO_3_S‐modified perovskite film, the PL peak shifts from 651.18 to 765.47 nm (Figure 3a–c and Figure S11). The initial PL peak of C_10_H_21_NaO_3_S‐modified perovskite film is located at 651.18 nm, different from the Control perovskite film and the NaCl‐modified perovskite film. Although all three samples showed a red shift in the PL peak, the C_10_H_21_NaO_3_S‐modified perovskite film exhibited a larger shift compared to the Control perovskite film and NaCl‐modified perovskite film. Meanwhile, the evolution of the PL full‐width at half‐maximum (FWHM) indicates that the nanocrystalline nuclei gradually grew and tended to homogenize. The FWHM of the Control perovskite film (70 nm → 50 nm), the NaCl‐modified perovskite film (83 nm → 50 nm), and the C_10_H_21_NaO_3_S‐modified perovskite film (115 nm → 50 nm) all shrink significantly, indicating that the nanocrystalline nuclei gradually grew and tended to homogenize [37] (Figure S11). The initial FWHM of the C_10_H_21_NaO_3_S‐modified perovskite film is relatively large, which implies that there is a strong interaction between sulfonic acid groups and perovskite (Figure S11). These results suggest the coexistence of multiscale grains in the early nucleation stage, which may be the interaction between C_10_H_21_NaO_3_S and perovskite nanocolloids [26, 38, 39].

**FIGURE 3:**
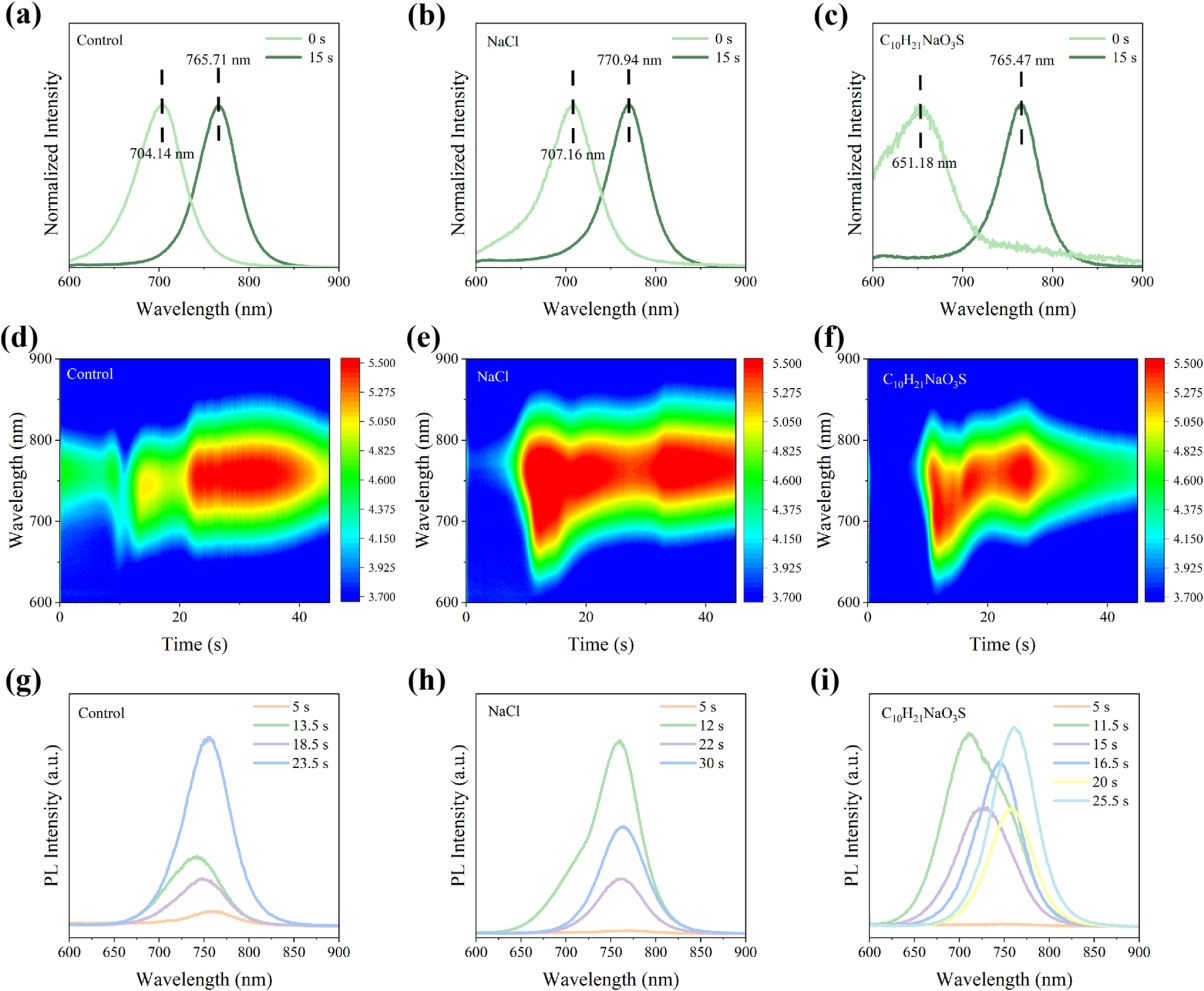
Shift of PL peak positions of (a) Control, (b) NaCl‐modified, and (c) C_10_H_21_NaO_3_S‐modified perovskite films when anti solvent is added dropwise. In situ PL during annealing of (d) Control, (e) NaCl‐modified and (f) C_10_H_21_NaO_3_S‐modified perovskite films. Changes in PL intensity of (g) Control, (h) NaCl‐modified, and (i) C_10_H_21_NaO_3_S‐modified perovskite films at different times during the annealing stage.

In situ photoluminescence (in situ PL) spectra acquired during the annealing process were systematically analyzed (Figure 3d–f). There are significant differences in the crystallization triggering times of the Control perovskite film, the NaCl‐modified perovskite film, and the C_10_H_21_NaO_3_S‐modified perovskite film. The PL intensity of the Control perovskite film jumps immediately after the start of annealing, indicating a rapid nucleation‐growth process. The PL intensity of the NaCl‐modified perovskite film increases with a delay of 6 s, while the PL intensity of the C_10_H_21_NaO_3_S‐modified perovskite film rises only after a delay of 8 s. This is because as the solvent volatilizes, more and more crystal nuclei are exposed. The nucleation rate of the C_10_H_21_NaO_3_S‐modified perovskite film is slightly slower than that of the Control perovskite film. The addition of C_10_H_21_NaO_3_S has the effect of delaying crystallization because of the interaction between the sulfonate ions and Pb^2+^, confirming that C_10_H_21_O_3_S^−^ delays nucleation through the chelating effect with Pb^2+^ [36]. The Control perovskite film and the NaCl‐modified perovskite film show a typical four‐stage ‘up–down–up–down’ process with two states [40] (Figure 3g–i and Figure S12). First, perovskite nanoparticles crystallize during the growth process, resulting in an increase of PL intensity. Then the solvent evaporates, exposing the surfaces of perovskite microcrystals to the surrounding environment, leading to the formation of grain boundaries and rapid quenching of PL. During the annealing process, smaller grains further grow and merge into larger grains, reducing the number of grain boundaries and leading to a further increase in PL intensity. Finally, the film is fragile under ambient conditions and easily decomposes. This leads to a stable PL intensity, but it is lower than the maximum PL [41]. Compared with the Control perovskite film and the NaCl‐modified perovskite film, the introduction of C_10_H_21_NaO_3_S induces a precrystallization process, which is manifested as an increase in PL intensity after the typical four‐stage ‘up–down–up–down’ process with two states. (Figure 3g–i and Figure S12) [40, 42]. After the PL intensity of C_10_H_21_NaO_3_S‐modified perovskite film decreased at 20s, the PL intensity increased again at 25.5 s (Figure 3i). This phenomenon is attributed to the coordination of C_10_H_21_O_3_S^−^ with Pb^2^^+^ [38, 43, 44], which prolongs the survival time of the precursor complex and promotes the ordered orientation growth. Compared with the Control and NaCl‐modified perovskite films, the C_10_H_21_NaO_3_S‐modified perovskite film exhibits a lower PL peak position and a larger FWHM in the first 8 s of the annealing stage, which indicates that there is a strong interaction between sulfonic acid groups and perovskite, which regulates the crystallization of perovskite (Figure S12). To better illustrate the role of sulfonic acid groups, we performed in‐situ PL on C_6_H_14_O_3_S‐modified perovskite film during annealing. Compared with the control perovskite film, the sulfonic acid group‐containing additive delays the crystallization process and optimizes crystallization kinetics. To study the interaction between C_10_H_21_NaO_3_S and PbI_2_, we monitored the changes in the absorption spectra of a PbI_2_ solution (Figure S13). After adding C_10_H_21_NaO_3_S, the concentration of PbI_3_^−^ increases more than adding DMSO. This implies that the interaction between C_10_H_21_NaO_3_S and PbI_2_ is stronger than that between DMSO and PbI_2_. The addition of C_10_H_21_NaO_3_S can disrupt the DMSO‐PbI_2_ solvated phase [45, 46]. These results indicate that the addition of C_10_H_21_O_3_S^−^ can improve film quality by regulating crystallization kinetics, which is consistent with the improved open‐circuit voltage (*V*_OC_) and fill factor (FF) of the corresponding device.

### 2.4 Characterization of Perovskite Films

The interaction between the perovskite film and C_10_H_21_NaO_3_S was further confirmed by performing XPS. Two characteristic peaks of Pb 4f (at 143and 138.1 eV) were observed for the C_10_H_21_NaO_3_S‐modified perovskite film; these peaks shifted toward lower binding energies by 0.45 and 0.4 eV, respectively (Figure 4a). Two characteristic peaks of S 2P were observed at 167.98 and 169.24 eV, corresponding to the binding energies of S 2P_3/2_ and S 2P_1/2_, respectively. After treatment with C_10_H_21_NaO_3_S, these characteristic peaks shifted towards the lower binding energy direction by 0.23 and 0.36 eV, respectively (Figure 4b). These suggest that the functional sulfonic acid group can interact with under‐coordination Pb^2+^. This interaction provides a strong passivation effect on defects in the perovskite film, effectively suppressing the nonradiative recombination. Similar low‐energy transitions were also observed in I 3d (Figure S14). After modification with C_10_H_21_NaO_3_S, the characteristic peaks I 3d_3/2_ and I 3d_5/2_ shifted toward lower binding energies by 0.3 eV. This confirms the strong hydrogen bond between C_10_H_21_NaO_3_S and I^−^, thereby inhibiting the migration of I^−^ [47]. In addition, the N 1s spectrum shifted by 0.32 eV, indicating the formation of S═O⋯ N between the organic cations of perovskite and the sulfonic acid groups of C_10_H_21_NaO_3_S [48]. Fourier transform infrared spectroscopy (FTIR) results show the characteristic stretching vibration peak of S═O in the full spectrum (Figure S15). This indicates a significant interaction between C_10_H_21_NaO_3_S and perovskite. This interaction originates from the undercoordinated Pb^2+^ and electron‐rich S═O groups [49].

**FIGURE 4:**
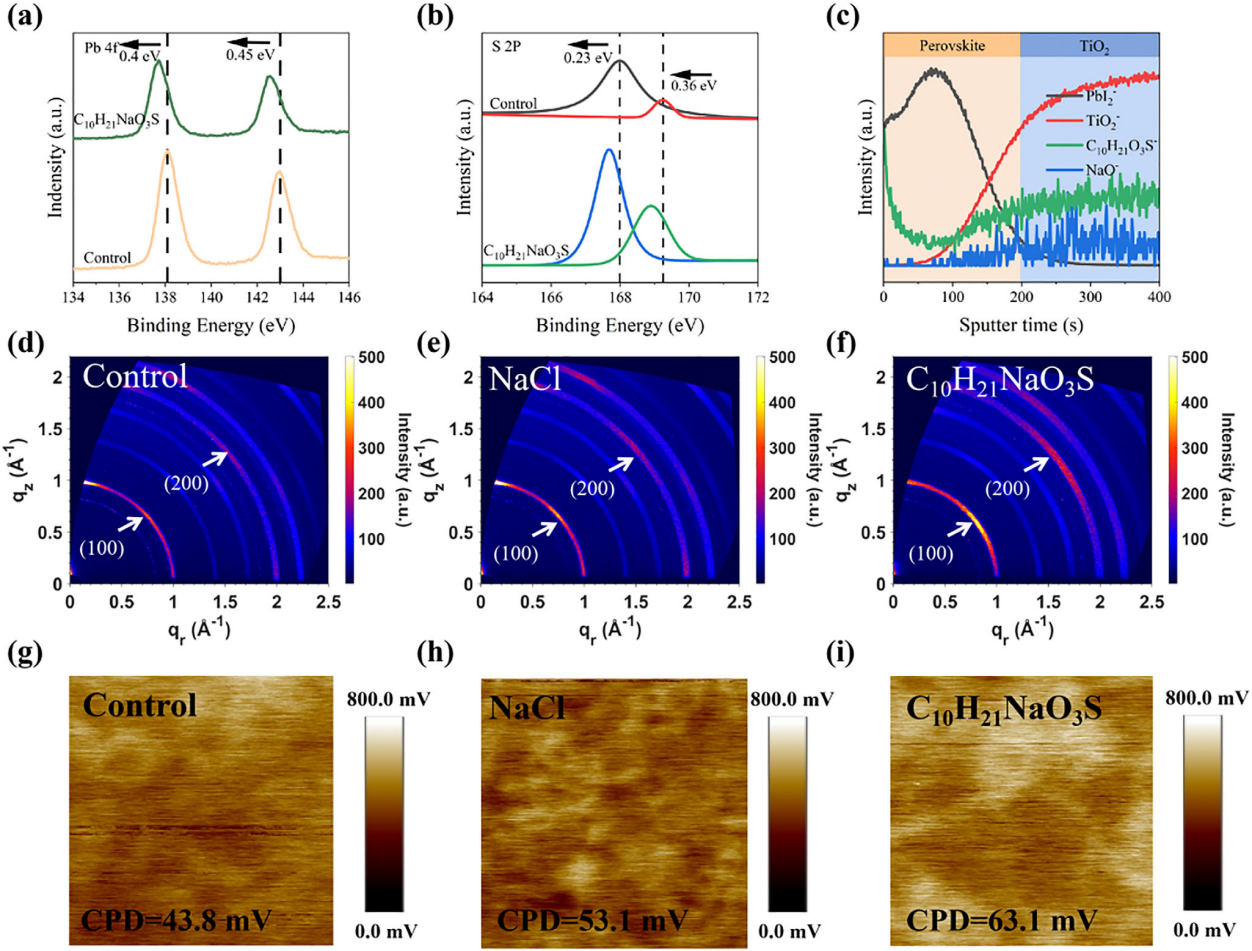
XPS spectra of Pb 4f (a) and S 2f (b) obtained from the surfaces of Control and C_10_H_21_NaO_3_S‐modified perovskite films. (c) TOF‐SIMS depth profile of perovskite with C_10_H_21_NaO_3_S. GIWAXS patterns of (d) Control, (e) NaCl‐modified and (f) C_10_H_21_NaO_3_S‐modified perovskite films. KPFM images of (g) Control, (h) NaCl‐modified and (i) C_10_H_21_NaO_3_S‐modified perovskite films.

We confirmed the spatial distribution of C_10_H_21_NaO_3_S in perovskite films through Time‐of‐Flight Secondary Ion Mass Spectrometry (ToF‐SIMS). The sulfonate groups are mainly distributed on the surface of the perovskite film, while Na^+^ is uniformly distributed within the perovskite film (Figure 4c). The in‐plane homogenization of perovskite films by Na^+^ doping motivated us to also probe the out‐of‐plane direction by evaluating the PL collected from both the front side (glass‐perovskite interface) and back side (perovskite‐air interface) of the sample. We plotted the normalized PL spectra for each sample, comparing measurements taken from the glass and air sides, and calculated the respective difference spectra (Figure S16). Additionally, PL mapping provides a direct analysis of the perovskite film's surface and reveals the distribution of PL intensity (Figure S17). The film with Na distribution shows a higher PL intensity and a more uniform PL distribution than the Control perovskite film. The PL intensity difference of perovskite film doped with 2% Na^+^ is reduced, indicating that the C_10_H_21_NaO_3_S‐modified perovskite film is more uniform.

To explore the influence of C_10_H_21_NaO_3_S on the crystallization of the perovskite film, we conducted grazing incidence wide‐angle x‐ray scattering (GIWAXS) analysis (Figure 4d–f). The perovskite displayed characteristic diffraction rings at scatter vector (q) values of 1, 1.37, 1.65, and 1.85 nm^−1^, related to the (100), (110), (111), and (200) crystal planes of perovskite films, respectively. With the addition of 2% Na^+^, the previously observed PbI_2_ signal weakened, and stronger reflections of the (100) and (200) planes were observed. This orientation regulation is beneficial for the transport of carriers, thereby improving the device performance. The XRD patterns of Control and C_10_H_21_NaO_3_S‐modified perovskite films at different annealing temperatures indicate that C_10_H_21_NaO_3_S significantly inhibits the precipitation of PbI_2_ (Figure S18). It reveals the regulatory effect of the C_10_H_21_NaO_3_S on the crystallization kinetics and orientation behavior of the perovskite film. This is attributed to the strain relaxation of the perovskite film caused by Na^+^ doping, the coordination effect between C_10_H_21_O_3_S^−^ and the Pb^2+^ octahedron, thus optimizing the stability of the perovskite lattice.

Next, the carrier dynamics of perovskite films were measured before and after optimization. Kelvin probe force microscopy (KPFM) measurements were performed (Figure 4g–i). The C_10_H_21_NaO_3_S‐modified perovskite films exhibit higher surface potentials than the Control and NaCl‐modified perovskite films, which is beneficial to the photogenerated carrier transport [50]. The steady‐state and time‐resolved photoluminescence (PL and TRPL) with and without the carrier transport layer demonstrate the enhancement of carrier extraction in C_10_H_21_NaO_3_S‐modified perovskite films (Figure S19). The PL intensity of the target (C_10_H_21_NaO_3_S‐modified) perovskite film increased compared to the Control perovskite film, suggesting that the addition of C_10_H_21_NaO_3_S significantly suppressed nonradiative recombination [51]. Subsequently, a hole transport layer (HTL) is deposited on the surface and perovskite is deposited on the electron transport layer (ETL) separately. The decrease in peak intensity is attributed to effective hole and electron extraction, leading to quenching of the fluorescence peak [52, 53, 54]. This indicates accelerated electron extraction and collection from perovskite to electron transport layer in C_10_H_21_NaO_3_S‐modified perovskite films [55, 56]. Besides, C_10_H_21_NaO_3_S‐modified perovskite film with a carrier transport layer shows faster decay of TRPL, which confirms more efficient charge transfer to the transport layer. We investigated the role of C_10_H_21_NaO_3_S in determining the carrier extraction process at the perovskite/HTL and ETL/perovskite interfaces with Transient Absorption Spectroscopy (TAS) decay dynamics [57, 58] (Figure S20). The kinetic decay curve of the C_10_H_21_NaO_3_S‐modified perovskite film decrease faster than that of the Control perovskite film for the ETL/perovskite interface. Therefore, after modification with C_10_H_21_NaO_3_S, it can accelerate the extraction of electrons without affecting the extraction of holes.

Ionic migration has been proven to be one of the key factors affecting the stability of PSCs [59], and alkali metal ions can effectively inhibit ion migration [60, 61]. Under illumination and electric field, mobile ions such as I^−^ migrate and accumulate in the bulk. Ions migrating to the interfaces damage the carrier transport layers, cause band bending and slow down the carrier transport. These effects ultimately lead to the continuous decay of the photoelectric conversion efficiency of the solar cell and a substantial reduction in its operational stability [59]. Ion migration within perovskite films can result in the formation of point defects. These defects destroy the perovskite film's electrical properties. Furthermore, under an applied electric field, the directional movement of ions and charged defects impairs the transport of photogenerated carriers and induces hysteresis [62]. We conducted in situ monitoring of the changes in perovskite thin films under external electric fields using PL mapping measurements (Figure S21). We observed photoluminescence quenching of the control film from the positive electrode to the negative electrode. This indicates that photoluminescence is suppressed by ion migration, resulting in the accumulation of high‐density defects in the dark region of the film [63]. After applying a 10 V electric field, the Control perovskite film exhibited a significant decrease in fluorescence intensity at the positive electrode side within the red circle, directly reflecting the ions migration. In contrast, the Na^+^‐doped perovskite film showed no obvious change in fluorescence intensity in the same region, indicating that the additive can effectively suppress ion migration at the positive electrode, thereby significantly improving the stability of the perovskite under an electric field. Therefore, we tested the activation energy of ion migration (Figure S22). The ion migration activation energy increases from 67.53 meV (Control perovskite film) to 84.39 meV (C_10_H_21_NaO_3_S‐modified perovskite film). This is attributed to the strong interaction between C_10_H_21_NaO_3_S and PbI_2_. A high ion migration activation energy can effectively inhibit the migration of Pb^2+^ and I^−^ ions and prevent the deterioration of device performance [64].

### 2.5 Application in Solar Cells

The optimized perovskite films were further fabricated as solar cell devices. To determine the optimal Na^+^ doping concentration that maximizes performance, systematic experiments were conducted on different sodium molar concentrations (Figure S23). The results indicate that the optimal performance is achieved when the sodium doping concentration is set to 2 mol%. This work stands out among its counterparts fabricated with an n‐i‐p architecture, demonstrating the reliability and competitiveness of our approach (Figure 5 and Table S1). The power conversion efficiency (PCE) of the Control device is 24.16%, the short‐circuit current density (*J*_SC_) is 25.74 mA/cm^2^, the open‐circuit voltage (*V*_OC_) is 1.16 V, and the fill factor (FF) is 80.83%. The device with the addition of NaCl (at the optimal concentration of 1 mg mL^−1^) yields a PCE of 25.06%. The device with the addition of C_10_H_21_NaO_3_S (at the optimal concentration of 1 mg mL^−1^) achieves the highest PCE of 26.67% (Figure 5a), mainly due to the increases in *V*_OC_ (1.19 V), FF (83.87%), and the current density (26.87 mA/cm^2^). The certificated efficiency is 26.44% (Figure S24). The statistical data of the performance parameters of the PSCs confirm the overall performance improvement of the C_10_H_21_NaO_3_S‐modified PSCs and demonstrate their excellent reproducibility (Figure 5b). The reliability of *J*_SC_ is verified by the external quantum efficiency (EQE) (Figure 5c). By analyzing the external quantum efficiency (EQE) spectrum of the target device, the bandgap of the target device is determined to be 1.53 eV, and this spectrum exhibits an inflection point at approximately 810 nm (Figure S25) [65].

**FIGURE 5:**
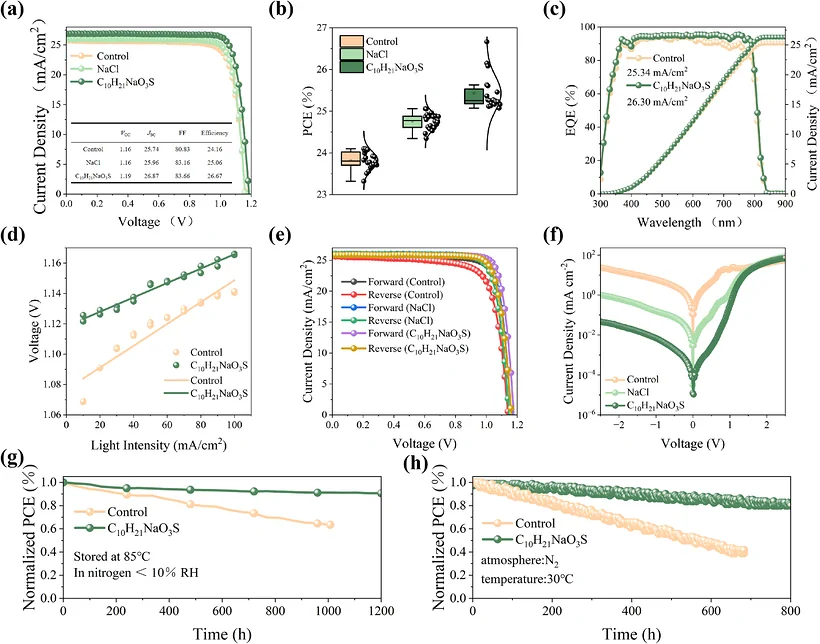
(a) *J*‐*V* curves of the champion solar cells under reverse scan. (b) PCE statistics of 20 PSCs. (c) External quantum efficiency (EQE) spectra and integrated photocurrent, integrated over the AM1.5 (100 mW cm^−2^) solar spectrum. (d) Linear relationship of *V*oc with the natural logarithm of light intensity. (e) The *J*‐*V* curves of the three devices are scanned forward and backward. (f) Dark *J*‐*V* curves. (g) Thermal stability of Control and C_10_H_21_NaO_3_S PSCs @85 °C. (h) Stabilized MPP tracking of N_2_‐encapsulated devices exposed to continuous 1‐sun illumination for 800 h at ambient temperature.

To understand the reason for the improvement of the fill factor (FF) in the C_10_H_21_NaO_3_S‐modified device, we conducted experiments on the open‐circuit voltage (*V*_OC_) under different light intensities to evaluate the charge carrier recombination mechanism of the device based on the ideality factor (*n*) (Figure 5d). Our results show that, as depicted in Figure 4d, the C_10_H_21_NaO_3_S‐modified device exhibits a lower n value of 1.15 kBT/q, while the Control device shows an n value of 1.42 k_B_T/q. This finding implies that the trap‐assisted Shockley–Read–Hall recombination in the C_10_H_21_NaO_3_S‐modified perovskite film is negligible and contributes to the increase in the fill factor [66, 67, 68]. In addition, we found that the incorporation of C_10_H_21_NaO_3_S significantly weakens the hysteresis phenomenon in the device (Figure 5e), attributed to the reduction of trap states and effective charge extraction [15, 69].

To further understand the reason for the observed higher *V*_OC_, we carried out capacitance‐voltage (C‐V) measurements (Figure S26) [70, 71]. The results indicate that the *V*_bi_ increases from 1.13 to 1.18 V. The increased *V*_bi_ confirms optimized carrier separation and transport pathways, contributing to achieve a higher *V*_OC_. The Shockley Equation is used to evaluate the device carrier transport and complex dynamics characteristics:

$$V_{\mathrm{OC}}=n\left(\text{KT}/q\right)\ln P_{\mathrm{light}}+C$$

where *n* is the ideal factor, *K* is the Boltzmann constant, *T* is the temperature, *q* is the elementary charge. *C* is a fitting parameter. As illustrated in the light‐intensity‐dependent *V*_OC_ curves, the linear extrapolation to zero light intensity yields a higher intercept for the target device (*C* = 1.12 V) compared to the control device (*C* = 1.08 V). This elevated intercept implies suppressed nonradiative recombination and minimized leakage current under low‐illumination limits.

The dark current is also a key factor to reflect the quality of PSCs. We performed the dark‐state *J*‐*V* curves of the Control, NaCl‐modified, and C_10_H_21_NaO_3_S‐modified devices, where the C_10_H_21_NaO_3_S‐modified device exhibits the smallest dark current density (1.05 × 10^−5^ mA/cm^2^) (Figure 5f). This further confirms that the interface defects/traps are effectively suppressed in the C_10_H_21_NaO_3_S‐modified device, enabling the efficient extraction of carriers without the formation of leakage currents, which is consistent with the higher open‐circuit voltage (*V*_OC_) [72]. To validate the defect reduction, the trap density in perovskite was comprehensively evaluated by fabricating electron‐only devices using the Space‐Charge Limited Current (SCLC) method (Figure S27) [73, 74, 75]. Compared to Control device, the *V*_TFL_ and *n*_trap_ of the NaCl‐modified and C_10_H_21_NaO_3_S‐modified devices were reduced. For electron‐only devices, *V*_TFL_ decreased from 0.30 to 0.225 V and 0.174 V, and *n*_trap_ decreased from 7.56 × 10^15^ to 5.67 × 10^15^ and 4.39 × 10^15^ cm^−3^. That suggests the composite additive‐modified perovskite devices have a reduced charge trap density. The current‐time (I‐T) curve of the PSC under continuous light irradiation for 600 s at the maximum power point (Figure S28). The stable output of the power conversion efficiency (PCE) of the C_10_H_21_NaO_3_S‐modified device within 600 s basically remains unchanged, indicating an improvement in stability. Electrochemical impedance spectroscopy (EIS) showed smaller series resistance (*R*_s_) and larger composite resistance (*R*_rec_) for the optimized device, signifying efficient carrier transmission (Figure S29) [76, 77]. As shown in Table S2, the C_10_H_21_NaO_3_S‐modified device exhibits a significantly higher combination resistance (*R*rec = 457.3 Ω) than the control device (*R*rec = 74.65 Ω), demonstrating a suppressed nonradiative recombination in the target device.

To study the stability of the device under accelerated aging conditions, the unencapsulated devices were aged under continuous heating at 85 ± 5°C in a N_2_‐filled glovebox (ISOS‐D‐2I, 85°C, N_2_). The target device shows a very small PCE decay (<10%) after 1,200 h, while the PCE decay of the Control device drops by nearly 40% (Figure 5g). We also examined the operational stability of the N_2_‐encapsulated devices under maximum power point (MPP) tracking at standard illumination according to ISOS‐L‐1 (with a room temperature of 30°C and a relative humidity of 30%–35%). After 800 h, the device treated with C_10_H_21_NaO_3_S still maintained 80% of its initial efficiency, while the efficiency of the Control device dropped below 40% (Figure 5h).

## 3 Conclusions

In this study, first‐principles calculations have been successfully used to design the Na^+^ doping for both α‐FAPbI_3_ and δ‐FAPbI_3_. The formation energies of Na^+^‐doped α‐FAPbI_3_ are much lower than those of δ‐FAPbI_3_ across all sites, enabling Na^+^ doping to effectively stabilize the α phase with higher stability.

As validated by experimental results, Na^+^ doping effectively relieves the residual strain of FAPbI_3_, lowers intrinsic defect density, and inhibits the unfavorable α to δ phase transition. Meanwhile, sulfonic acid groups optimize perovskite crystallization. Consequently, the thermal and photostability of the perovskite films are greatly improved.

The optimized film delivers a champion PCE of 26.67% in solar cells, with 80% efficiency retention after 800 h light soaking and 90% retention after 1200 h aging at 85°C. This work offers a new strategy of lattice engineering in perovskite crystals and devices.

## Author Contributions

**Zhihuan Tang**: writing – original draft, visualization, data curation, investigation, methodology. **Mingzi Sun**: investigation, validation. **Jinghao Ge**: supervision, investigation. **Ying Wang**: investigation, validation. **Yunfan Wang**: investigation, validation, formal analysis. **Yiran Tao**: investigation, validation. **Lu Zhang**: writing – review and editing, formal analysis, supervision. **Xinhui Lu**: supervision, investigation. **Sai‐Wing Tsang**: supervision, investigation. **Weizhong Tian**: investigation. **Rui Wang**: validation. **Hao‐Chung Kuo**: resources. **Bolong Huang**: supervision, investigation, software, validation. **Shengzhong (Frank) Liu**: writing – review and editing, resources, project administration. **Jiaxue You**: supervision, writing – review and editing, funding acquisition, conceptualization, methodology, project administration. **Alex K.Y. Jen**: supervision.

## Conflicts of Interest

The authors declare no conflicts of interest.

## Supporting information




**Supporting File**: anie73967‐sup‐0001‐SuppMat.docx.

## Data Availability

The data that support the findings of this study are available from the corresponding author upon reasonable request.
