# Supplementary material for: Mitigating Stress‐Induced Nonphotoactive Phase Transition Through Sodium Sulfonate Engineering for Stable and Efficient Perovskite Solar Cells
Source: Angew Chem Int Ed Engl. 2026 Aug 5;65(40):e2425823. doi: 10.1002/anie.2425823 (PMC13618294; doi:10.1002/anie.2425823)
Supplement: Supplementary file 1 [file ANIE-65-e2425823-s001.docx]

**Supporting Information for**

**Mitigating Stress-Induced Non-Photoactive Phase Transition through Sodium Sulfonate Engineering for Stable and Efficient Perovskite Solar Cells**

Zhihuan Tang^1^, Mingzi Sun^2^, Jinghao Ge^1^, Ying Wang^1^, Yunfan Wang^3^, Yiran Tao^4^, Lu Zhang^1^, Xinhui Lu^4^, Sai‐Wing Tsang^3^, Weizhong Tian^5^, Rui Wang^5^, Hao-Chung Kuo^6^, Bolong Huang^2*^, Shengzhong (Frank) Liu^1,7,8*^, Jiaxue You^3*^, Alex K.Y. Jen^2,3^.

^1^Institute for Advanced Energy Materials, School of Materials Science and Engineering, Shaanxi Normal University, Xi’an 710119, China.

^2^Department of Chemistry, City University of Hong Kong, Kowloon, Hong Kong SAR, China.

^3^Department of Materials Science and Engineering, Hong Kong Institute for Clean Energy, City University of Hong Kong, Hong Kong SAR, China.

^4^Department of Physics, The Chinese University of Hong Kong, Shatin 999077, Hong Kong SAR, China.

^5^Department of Materials Science and Engineering, School of Engineering, Zhejiang Provincial Key Laboratory of Intelligent Low-Carbon Biosynthesis, Westlake University, Hangzhou, China

^6^Semiconductor Research Center, Hon Hai Research Institute, Taipei, Taiwan, China.

^7^Key Laboratory of Photoelectric Conversion and Utilization of Solar Energy, Dalian Institute of Chemical Physics, Chinese Academy of Sciences, Dalian, 116023, Liaoning, China.

^8^Center of Materials Science and Optoelectronics Engineering, University of Chinese Academy of Sciences, Beijing 100049, China.

Corresponding authors*: BH: [b.h@cityu.edu.hk](mailto:b.h@cityu.edu.hk), SL: szliu@dicp.ac.cn, JY: jiaxuyou@cityu.edu.hk.

**Figure S1-S29**

Figure S1. The structures of C_10_H_21_NaO_3_S.
Figure S2. The electronic distributions of (a) α-FAPbI_3_ and (b) δ-FAPbI_3_. Dark grey balls = Pb, brown balls = I, grey balls = C, blue balls = N, and white balls = H. The blue and green isosurfaces represent the bonding and anti-bonding orbitals near the EF, respectively. The PDOS of (c) α-FAPbI_3_ and (d) δ-FAPbI_3_.
Figure S3. The relaxed structures of the δ-FAPbI_3_ with different doping sites. (a) IntNa-1, (b) IntNa-2, (c) IntNa-3, and (d) SubNa. Dark grey balls = Pb, brown balls = I, purple balls = Na, grey balls = C, blue balls = N, and white balls = H.
Figure S4. High-resolution transmission electron microscopy (HRTEM) of the Control and C_10_H_21_NaO_3_S-modified perovskite films.
Figure S5. Free-energy calculation for the α-to-δ transition of FAPbI3 perovskites with or without Na^+^ doping.
Figure S6. Control perovskite film and NaCl-modified perovskite film after different illumination times (On the left side of the picture is Control perovskite film, and on the right side is NaCl-modified perovskite film).
Figure S7. HR-TEM images and TEM EDS mappings of perovskite grains.
Figure S8. Full XPS spectra of FAPbI_3_, in which the peak of Na 1s was found at~1071 Ev.
Figure S9. XRD reflections of the (100) plane of perovskite films containing different amounts of Na^+^.
Figure S10. In situ PL during spin-coating of (d) Control, (e) NaCl-modified and (f) C_10_H_21_NaO_3_S-modified perovskite films.
Figure S11. Movement of (a) PL peak positions and (b) FWHM of Control, NaCl-modified and C_10_H_21_NaO_3_S-modified perovskite films when antisolvents are added dropwise (Starting from the time when the anti solvent drips).
Figure S12. Movement of (a) PL peak positions, (b) FWHM and (c) PL intensity of Control, NaCl-modified and C_10_H_21_NaO_3_S-modified perovskite films during the annealing stage. (d) In situ PL during annealing of C_6_H_14_O_3_S-modified perovskite films.
Figure S13. UV-vis absorption spectra of PbI_2_ in DMF.
Figure S14. XPS spectra of I 3d (a) and N 1s (b) obtained from the surfaces of Control and C_10_H_21_NaO_3_S-modified perovskite films.
Figure S15. The FTIR spectra in the regions of 1000-1100 cm^−1^ for C_10_H_21_NaO_3_S@FAI films.
Figure S16. Steady-state PL results (normalized) from glass and air sides of perovskite films without and with 2%mol Na^+^.
Figure S17. PL mapping images for emission intensity.
Figure S18. Temperature dependent XRD reflection of Control perovskite film and C_10_H_21_NaO_3_S-modified perovskite film.
Figure S19. PL curves (a-c) of Control and C_10_H_21_NaO_3_S-modified perovskite films with and without a carrier transport layer. TRPL curves (d-f) of Control and C_10_H_21_NaO_3_S-modified perovskite films with and without a carrier transport layer.
Figure S20. Transient absorption spectroscopy (TAS) kinetics for Control and C_10_H_21_NaO_3_S-modified perovskite films with and without a carrier transport layer.
Figure S21. PL mapping of perovskite films under external electric fields.
Figure S22. Arrhenius plots of the characteristic transition frequencies determined from the derivative of the admittance spectra of a) Control, b) C_10_H_21_NaO_3_S-modified perovskite films.
Figure S23. (a) *V*_OC_, (b) FF and (c) PCE statistics of 20 PSCs.
Figure S24. certificated efficiency.
Figure S25. The curve of the first derivative of quantum efficiency (EQE) as a function of wavelength.
Figure S26. Mott-Schottky plots.
Figure S27. SCLC plots of electron-only devices.
Figure S28. Steady-state output during measurements of the photocurrent density and PCE.
Figure S29. Electrochemical impedance spectroscopy (EIS) curves of PSCs.

**Table S1-S2**

Table S1. Photovoltaic parameters of n-i-p perovskite solar cells.
Table S2. EIS fitting parameters of the PSCs.

**Experimental Section**

**Materials**

Sodium decane-1-sulfonate (C_10_H_21_NaO_3_S，98%) were purchased from Bidepharm. NaCl and C_10_H_21_NaO_3_S were purchased from Bidepharm. Dimethyl sulfoxide (DMSO, purity 99.9%) and N,N-dimethylformamide (DMF, purity 99.8%) were purchased from Aladdin Biochemical Technology Co., Ltd. 4-tert-butylpyridine (t-BP), ethyl acetate (EAC), diethyl ether, isopropanol (IPA), and lithium bis(trifluoromethylsulfonyl) imide salt (Li-TFSI) were purchased from Sigma-Aldrich. Lead(II) iodide (PbI₂, purity 99.999%), Formamidinium iodide (FAI, purity 99.9%), methylammonium chloride (MACl, purity 99.99%), methylammonium iodide (MAI, purity 99.9%), methylammonium bromide (MABr, purity 99.9%), cesium iodide (CsI, purity 99.9%), and spiro-OMeTAD were purchased from Liaoning Youxuan New Energy Technology Co., Ltd. Methylammonium chloride (MACl, 99.99%), Poly[bis(4-phenyl)(2,4,6-triMethylphenyl)aMine] (PTAA) and 2-Fluorophenylethyl iodide (o-F-PEAI) were purchased from Xi’an Yuri Solar Co., Ltd.

**Solution preparation**

Perovskite precursor solution was obtained by combining PbI_2_ (1.2 mmol, 553.2 mg), FAI (1.2 mmol, 206.3 mg), and MACl (0.42 mmol, 28.3 mg) in 1 mL mixed solvent (DMF:DMSO = 4:1) to yield FAPbI_3_. Additives (NaCl and C_10_H_21_NaO_3_S) were added into the precursor at 1 mg/mL. For Spiro-OMeTAD solution, 90 mg Spiro-OMeTAD, 22 μL Li-TFSI solution (520 mg in 1 mL acetonitrile) and 36 μL 4-tert-butylpyridine were dissolved in 1 mL chlorobenzene (CB). The solutions were stirred at room temperature for 12 h and then filtered through a 0.45-μm syringe filter.

**Device fabrication**

Fluorine-doped tin oxide (FTO) glass was chosen as the substrate. The FTO was cleaned by ultra-sonication sequentially in cleaning concentrate, deionized water, and ethanol for 30 min. Then the cleaned substrate was dried by nitrogen flow and treated in a UV-ozone cleaner for 15 min. The TiO_2_ electron-transfer layer was prepared by chemical-bath deposition on FTO/glass substrate as reported previously. Firstly, the cleaned FTO/glass substrate was treated with UV-ozone cleaner for 10 min. Next, the substrate was placed in the 0.2-M TiCl_4_ aqueous solution at 70 °C for 60 min, and then the substrate was washed with deionized water and ethyl alcohol alternately three times. The substrate was then annealed at 200 °C for 30 min in air and treated in UV-ozonefor 20 min to enhance the wettability. Approximately 80 μL perovskite precursor was dropped onto the TiO_2_/FTO/glass substrates, and the anti-solution method was employed to form perovskite films with diethyl ether as the anti-solvent. The solution was spin-coated at 3000 rpm for 10 s, and then at 5000 rpm for 30 s. Twenty seconds before the end of the 5000-rpm step, 600 μL of diethyl ether was quickly dropped onto the surface. Next, the film was annealed at 150 °C for 15 min. After the PVK film was cooled, PEAI solution (5 mg/ml in IPA) was spin-coated on the PVK film at 2000 rpm for 30 s for top surface passivation without thermal annealing. Then, 50 μL Spiro OMeTAD solution was spin-coated at 5000 rpm for 30 s to form the hole-transfer layer. The gold electrode (~80 nm) was thermally evaporated onto the surface of the Spiro OMeTAD through a shadow mask, and the active area is 0.09 cm2. For the thermal stability of devices, the perovskite precursor solution was obtained by combining PbI_2_ (1.60 mmol, 735.3 mg), FAI (1.31 mmol, 224.4 mg), MABr (0.15 mmol, 16.2 mg), and CsI (0.08 mmol, 19.8 mg) with 5% excess of PbI_2_ in 1 mL mixed solvent (DMF:DMSO = 4:1) to yield Cs_0.05_FA_0.85_MA_0.10_Pb(I_0.97_Br_0.03_)_3_. 12 mg PTAA was dissolved in 1 ml toluene, then 6 μL Li-TFSI (340 mg Li-TFSI in 1 mL acetonitrile) and 6 μL tBP were added. The PTAA solution was spin-coated on surface of the perovskite layer at 2000 rpm. for 30 s. Finally, an 80-nm Au electrode was vapor deposited on the top of the Spiro-OMeTAD layer. For the p-i-n structured devices, 1.4 M perovskite precursor solution with the composition of (FA_0.95_MA_0.05_)_0.95_Cs_0.05_Pb(I_0.95_Br_0.05_)_3_ was prepared by fully dissolving corresponding CsI and FAI, MABr, PbBr_2_, PbI_2_ in a mixed solvent of DMF and DMSO with a volume ratio of 4:1. Additional 25 mol% of MACl were added into the precursor solution for better crystallization and perovskite phase transformation. The precursor solution was stirred for at least 6 hours, then filtered by using a syringe and a 0.45 μm filter before spinning coating. The HTLs precursor was spin-coated at 5000 rpm for 30 s on the substrate, and the obtained HTLs substrates were annealed at 100 ℃ for 10 minutes. The ITO/HTLs substrates did not require any treatment. Then, the perovskite precursor solution was spin coated at 2000 rpm for 2 s, 4000 rpm for 40 s. At the 10th second by the end of the progress, 150 μL CB was evenly dripped onto the substrate. Then the substrate was annealed at 100 ℃ for 30 min to form the perovskite layer. An interfacial passivation layer was formed by spin-coating PDADI solution (0.5 mg mL^-1^ in IPA) onto perovskite films at 3000 rpm for 30 s, followed by thermal annealing at 100°C for 9 min. PCBM (20 mg mL^-1^ in CB) was spin-coated on perovskite films at 2000 rpm for 30 s. BCP solution (0.5 mg mL^-1^ in isopropanol) was then dynamically spin-coated on the PCBM layer at 5000 rpm for 30 s. Finally, a 80 nm thick Ag electrode was deposited on the top of the BCP layer by thermal evaporation to complete the device. The device’s active area is 0.09 cm^2^.

**Device characterization**

Fourier transform infrared spectroscopy (FTIR) was recorded on Bruker EQUINX55

spectrometer. XPS and UPS analyses were performed using a photoelectron spectrometer (ESCALAB 250Xi, Thermo Fisher Scientific). UV-vis absorption spectra were recorded on a PerkinElmer Lambda 950 recording spectrophotometer. Grazing-incidence X-ray diffraction (GIXRD) spectra were recorded by a PANalytical Empyrean (Netherlands).

A field-emission scanning electron microscope (FE-SEM, JEOL SU-8020) was used toacquire the surface morphology image of perovskite films. The steady-state photoluminescence (PL) spectrum was obtained by FLS980 spectrometer (Edinburgh Instruments Ltd) with the excitation wavelength of 510 nm. Time-resolved PL (TRPL) spectra were measured with a PicoQuant FluoQuant 300. The samples for PL and TRPL were prepared on glass. The *J-V* curve was also captured with Keithley 2400 source but under AM 1.5 G illumination at 100 mW/cm^2^. The space-charge limited current (SCLC) measurement and dark *J-V* test were performed using Keithley 2400 source measurement unit under dark conditions. The external quantum efficiency (EQE) of the PSCs was recorded by QTest Station 2000ADI EQE measurement system (Crowntech Inc.). A reference silicon detector was calibrated to adjust the monochromatic light intensity. The CV and EIS measurement was stimulated by the Zahner electrochemical workstation (IM6ex, Germany). ToF-SIMS measurement was performed on a PHI nanoTOF II ToF-SIMS instrument.

**Calculation Setup**

In this work, density functional theory (DFT) calculations are carried out using the CASTEP package^[1]^. The Perdew-Burke-Ernzerhof (PBE) functional within the generalized gradient approximation (GGA) is employed to accurately describe exchange-correlation effects^[2-4]^. Ultrasoft pseudopotentials are used with a plane-wave cutoff energy of 410 eV. The geometry optimizations are performed using the BFGS algorithm with coarse k-point sampling ^[5]^. Convergence criteria are set to 0.001 eV/Å for atomic forces and 5×10^-5^ eV/atom for total energy changes during geometry optimizations. For the perovskite materials, we have applied the 2×2×2 supercells for both α-FAPbI_3_ and δ-FAPbI_3_ with the introduction of Na^+^ doping in different positions.

Figure S1. The structure of C_10_H_21_NaO_3_S.

Stress-induced non-photoactive phase transition is a critical factor limiting the stability of perovskite solar cells. Relevant studies have confirmed that introducing small-sized cations or applying appropriate compressive strain can effectively suppress the transformation of perovskite from α-FAPbI_3_ to the δ-FAPbI_3_, thereby enhancing phase stability and device lifetime. Alkali metal cations have been widely employed for lattice regulation and defect passivation in perovskites, among which the mechanism of K^+^ has been systematically investigated^[6-9]^. Compared with K^+^, Na^+^ possesses a smaller ionic radius and higher charge density, enabling it to bind more effectively with defect sites in the perovskite lattice, better stabilize the lattice structure, and inhibit ion migration. Based on the above ionic characteristics and molecular design strategy, this work selects Na^+^-containing sodium 1-decanesulfonate (C_10_H_21_NaO_3_S) as an additive to realize the synergistic regulation of stress, crystallization and defects in perovskite films.

Figure S2. The electronic distributions of (a) α-FAPbI_3_ and (b) δ-FAPbI_3_. Dark grey balls = Pb, brown balls = I, grey balls = C, blue balls = N, and white balls = H. The blue and green isosurfaces represent the bonding and anti-bonding orbitals near the EF, respectively. The PDOS of (c) α-FAPbI_3_ and (d) δ-FAPbI_3_.

First of all, we have compared the electronic distributions regarding the bonding and anti-bonding orbitals near the Fermi level (E_F_) for both α-FAPbI_3_ and δ-FAPbI_3_. For the α-FAPbI_3_, the I-5p and Pb-6p orbitals dominate the bonding and anti-bonding orbitals, respectively (Figure S2a). The FA does not contribute to the electronic distributions near the E_F_. In contrast, such distributions have been changed for the δ-FAPbI_3_, where the bonding orbitals still mainly concentrate on the I sites, while the anti-bonding orbital distribution is noted on the FA^+^ rather than the Pb^2+^ sites (Figure S2b). This indicates that the phase changes of FAPbI_3_ not only affect the stability but also the electronic structures, which further affect their optoelectronic performances. To further understand the electronic structures, we have demonstrated the projected partial density of states (PDOS) for the perovskite materials. Notably, the valence band maximum (VBM) and conduction band minimum (CBM) of the α-FAPbI_3_ are constituted by the I-5p and Pb-6p orbitals, respectively (Figure S2c). There is a bandgap of 1.6 eV for the α-FAPbI_3_, which is smaller than that of the δ-FAPbI_3_ (3.2 eV) and supplies the higher optical activity for the α-FAPbI_3_. With the phase change to the δ-FAPbI_3_, the Pb-orbitals above the E_F_ upshift, leading to the FA-2p orbitals becoming the main contributions to the CBM (Figure S2d). Meanwhile, the I-5p orbitals show a converse trend, where the orbitals below the EF have downshifted, which further affects the electronic structures of the δ-FAPbI_3_.

Figure S3. The relaxed structures of the δ-FAPbI_3_ with different doping sites. (a) IntNa-1, (b) IntNa-2, (c) IntNa-3, and (d) SubNa. Dark grey balls = Pb, brown balls = I, purple balls = Na, grey balls = C, blue balls = N, and white balls = H.

Figure S4. High-resolution transmission electron microscopy (HRTEM) of the Control and C_10_H_21_NaO_3_S-modified perovskite films.

Figure S5. Free-energy calculation for the α-to-δ transition of FAPbI_3_ perovskites with or without Na^+^ doping.

Figure S6. Control perovskite film and NaCl-modified perovskite film after different illumination times (On the left side of the picture is Control perovskite film, and on the right side is NaCl-modified perovskite film).

After subjecting the Control perovskite film and NaCl-modified perovskite film to constant 1-sun illumination for 158 h, a yellow phase (δ-FAPbI_3_) appeared on the Control perovskite film, while the NaCl-modified perovskite film remained in the black phase (α-FAPbI_3_).

Figure S7. HR-TEM images and TEM EDS mappings of perovskite grains.

Figure S8. Full XPS spectra of FAPbI_3_, in which the peak of Na 1s was found at~1071 Ev.

Full X-ray photoelectron spectroscopy (XPS) confirmed the existence of Na element over FAPbI_3_ with a Na 1s peak (1071 eV).

Figure S9. XRD reflections of the (100) plane of perovskite films containing different amounts of Na^+^.

The addition of Na^+^ ions caused a shift in the XRD peaks toward higher angles.

Figure S10. In situ PL during spin-coating of (d) Control, (e) NaCl-modified and (f) C_10_H_21_NaO_3_S-modified perovskite films.

In the initial stage of spin coating (~0-5 s), the precursor wet film did not form an ordered crystal phase due to solvation effect, so no obvious PL signal was detected. When an antisolvent is introduced, the solution quickly reaches a supersaturated state, triggering nucleation, at which point the initial PL emission of nanocrystalline nuclei can be observed.

Figure S11. Movement of (a) PL peak positions and (b) FWHM of Control, NaCl-modified and C_10_H_21_NaO_3_S-modified perovskite films when antisolvents are added dropwise (Starting from the time when the anti solvent drips).

Figure S12. Movement of (a) PL peak positions, (b) FWHM and (c) PL intensity of Control, NaCl-modified and C_10_H_21_NaO_3_S-modified perovskite films during the annealing stage. (d) In situ PL during annealing of C_6_H_14_O_3_S-modified perovskite films.

Figure S13. UV-vis absorption spectra of PbI_2_ in DMF.

Figure S14. XPS spectra of I 3d (a) and N 1s (b) obtained from the surfaces of Control and C_10_H_21_NaO_3_S-modified perovskite films.

After modification with C_10_H_21_NaO_3_S, the characteristic peaks I 3d3/2 and I 3d5/2 shifted towards lower binding energies by 0.3 eV. This confirms the strong hydrogen bond between C_10_H_21_NaO_3_S and I-, thereby inhibiting the migration of I-. In addition, the N 1s spectrum shifted by 0.32 eV, indicating the formation of S=O ··· N between the organic cations of perovskite and the sulfonic acid groups of C_10_H_21_NaO_3_S.

Figure S15. The FTIR spectra in the regions of 1000-1100 cm^−1^ for C_10_H_21_NaO_3_S@FAI films.

After the addition of FAI, the S=O tensile vibration peaks on the C_10_H_21_NaO_3_S shifted 11.7 cm^−1^ to the low wavenumber. This indicates a significant interaction between C_10_H_21_O_3_S^-^ and perovskite. After the addition of PbI_2_, the S=O tensile vibration peaks on the C_10_H_21_NaO_3_S shifted 15.4 cm^−1^ to the low wavenumber. This indicates a significant interaction between C_10_H_21_O_3_S^-^ and PbI_2_.

Figure S16. Steady-state PL results (normalized) from glass and air sides of perovskite films without and with 2%mol Na^+^.

We plotted the normalized PL spectra of each sample, compared the measured values on the glass and air sides, and calculated their respective difference spectra. The PL intensity difference of the perovskite film doped with 2% Na^+^ is reduced compared to the Control perovskite film, indicating that the C_10_H_21_NaO_3_S-modified perovskite film is more uniform.

Figure S17. PL mapping images for emission intensity.

Figure S18. Temperature dependent XRD reflection of Control perovskite film and C_10_H_21_NaO_3_S-modified perovskite film.

We conducted XRD measurements of perovskite films with the Control perovskite film and C_10_H_21_NaO_3_S-modified perovskite film at different temperatures, which showed that PbI_2_ was significantly inhibited in the C_10_H_21_NaO_3_S-modified perovskite film.

Figure S19. PL curves (a-c) of Control and C_10_H_21_NaO_3_S-modified perovskite films with and without a carrier transport layer. TRPL curves (d-f) of Control and C_10_H_21_NaO_3_S-modified perovskite films with and without a carrier transport layer.

Figure S14a is steady-state PL spectra of the Control and C_10_H_21_NaO_3_S-modified perovskite films on a glass substrate, respectively. C_10_H_21_NaO_3_S-modified perovskite films have the highest steady-state PL intensity, which indicates that C_10_H_21_NaO_3_S greatly inhibits non-radiative recombination caused by defects at grain boundaries. For glass/TiO_2_/perovskite structures, the PL intensity of the C_10_H_21_NaO_3_S-modified perovskite film is significantly reduced compared to Control perovskite film, due to stronger fluorescence quenching in C_10_H_21_NaO_3_S-modified perovskite film (Figure S11b). For glass/perovskite/spiro structures, the PL intensity of the C_10_H_21_NaO_3_S-modified perovskite film is no significant difference between Control perovskite film and C_10_H_21_NaO_3_S-modified perovskite film (Figure S11c). Figure S11 d-f is steady-state TRPL spectra of the Control and C_10_H_21_NaO_3_S-modified perovskite films for glass/perovskite structures, glass/TiO_2_/perovskite structures and glass/perovskite/spiro structures, respectively. For glass/perovskite structures, C_10_H_21_NaO_3_S-modified perovskite film shows slower decay. For glass/perovskite/spiro structures and glass/TiO_2_/perovskite structures, C_10_H_21_NaO_3_S-modified perovskite film Shows faster decay

Figure S20. Transient absorption spectroscopy (TAS) kinetics for Control and C_10_H_21_NaO_3_S-modified perovskite films with and without a carrier transport layer.

Transient absorption (TA) spectra was used to study the modification of C_10_H_21_NaO_3_S in carrier extraction at the TiO_2_/perovskite transport layer (ETL) interface transport layer and perovskite/spiro transport layer (HTL) interface. Compared with the Control perovskite film, the modification of C_10_H_21_NaO_3_S can accelerate the extraction and collection of the electron from perovskite to ETL transport layer.

Figure S21. PL mapping of perovskite films under external electric fields.

To realize the changes in perovskite films under external electric fields via PL mapping measurements, lateral structured devices with an Ag/perovskite/Ag configuration were fabricated, featuring a channel width of 150 μm. A 532 nm continuous-wave laser was adopted as the excitation light source. A 50× objective lens (Zeiss LD EC Epiplan-Neofluar, numerical aperture = 0.55) was employed to focus the laser beam onto the film surface and simultaneously capture the photoluminescence signals. Spatial-resolved PL information was collected by horizontally translating the microscope stage (along the X and Y axes) to record a series of discrete PL spectra, which were further processed to reconstruct 2D PL mapping images. A charge-coupled device was utilized for PL spectral detection. The scanning area was defined as 200 μm × 100 μm, and the spectral detection was centered at 810 nm. With an applied bias of 10 V on the device, the PL maps were recorded.

Figure S22. Arrhenius plots of the characteristic transition frequencies determined from the derivative of the admittance spectra of a) Control, b) C_10_H_21_NaO_3_S-modified perovskite films.

Conductivity was measured in the dark over a range of temperatures, and Ea was extracted from the data using the Arrhenius equation:

$\text{f}_{\text{0}}\text{ = }\text{f}_{\text{0}}^{\text{0}}\text{exp(}\frac{\text{-}\text{E}_{\text{a}}}{\text{kT}}\text{)}$,

Where $\text{f}_{\text{0}}$ is the characteristic relaxation frequency (extracted from admittance spectroscopy),$\text{f}_{\text{0}}^{\text{0}}$ is the pre-exponential factor (frequency constant), T denotes the temperature, and k denotes the Boltzmann constant. The range of T is from -80 K to 100 K. Take the natural logarithm of both sides of the Arrhenius equation to convert the exponential relationship into a linear relationship:

$\ln\text{f}_{\text{0}}\text{ = }\ln\text{f}_{\text{0}}^{\text{0}}\text{-}\frac{\text{E}_{\text{a}}}{\text{kT}}$,

Corresponding to the axes in the figure: Vertical axis (Y): $\ln\text{f}_{\text{0}}\text{ }$ (natural logarithm of frequency), Horizontal axis (X): $\frac{\text{1000}}{\text{T}}$, Linear standard form: $\text{Y = A+BX}$, where the intercept $\text{A = }\ln\text{f}_{\text{0}}^{\text{0}}$ and the slope $\text{B = -}\frac{\text{1000}\text{E}_{\text{a}}}{\text{k}}$. For the admittance spectrum at each temperature, calculate the derivative of conductance/capacitance with respect to frequency to obtain a derivative-frequency curve. The frequency corresponding to the peak of the derivative curve is the characteristic relaxation frequency ($\text{f}_{\text{0}}$) at that temperature. The activation energy $\text{E}_{\text{a}}$ can be obtained from the fitted slope B:

$$\text{E}_{\text{a}}\text{ = -B×1000×k}$$

The standard error of slope of Control is 0.12975, and that for the C_10_H_21_NaO_3_S is 0.14077. The coefficient of determination (R^2^) of Control is 0.92402, and that for the C_10_H_21_NaO_3_S is 0.94163.

Figure S23. (a) *V*_OC_, (b) FF and (c) PCE statistics of 20 PSCs.

The statistical data of the performance parameters of the PSCs confirm the overall performance improvement of the C_10_H_21_NaO_3_S-modified PSCs and demonstrate their excellent reproducibility.

Figure S24. **C**ertificated efficiency.

The C_10_H_21_NaO_3_S-modified perovskite solar cells (optimal concentration of 1 mg mL^-1^) obtained 26.44% PCE, 1.19V *V*_OC_, 83.23% FF, and 26.74 mA/cm^2^ current density.

Figure S25. The curve of the first derivative of quantum efficiency (EQE) as a function of wavelength.

Figure S26. Mott-Schottky plots.

The built-in voltage (V_bi_) of C_10_H_21_NaO_3_S-modified device is 1.18 V, which is higher than the Control device (1.13 V). The fitting is based on the depletion layer capacitance model of Schottky junction for n-type perovskite solar cells, with the core Mott–Schottky equation:

$$\frac{\text{1}}{\text{C}^{\text{2}}}\text{ = }\frac{\text{2(}\text{V}_{\text{bi}}\text{-V)}}{\text{qε}\text{ε}_{\text{0}}\text{A}^{\text{2}}\text{N}_{\text{d}}}$$

Where C is depletion layer capacitance of the device, V is applied DC bias voltage, $\text{V}_{\text{bi}}$ is built-in potential, q is elementary charge, $\text{ε}$ is relative dielectric constant of perovskite, $\text{ε}_{\text{0}}$ is vacuum permittivity, A is effective photosensitive area of the device, $\text{N}_{\text{d}}$ is donor carrier concentration in the perovskite film.

The linear region is selected as the plateau segment where $\frac{\text{1}}{\text{C}^{\text{2}}}$ decreases linearly with voltage, corresponding to the voltage range of 0.2 V to 1.0 V in the figure. Extrapolate the linearly fitted straight line to the horizontal voltage axis. The intercept voltage at the intersection of the line and the horizontal axis $\frac{\text{1}}{\text{C}^{\text{2}}}\text{ = }$0 is defined as the built-in potential $\text{V}_{\text{bi}}$.

Figure S27. SCLC plots of electron-only devices.

The dark *J*–*V* curves feature three distinct parts: the ohmic region, the trap-filling limited (TFL) region, and the trap-free child's region. The trap density (nt) is derived from the trap filling limited voltage (*V*_TFL_) at the intersection of the ohmic region and the trap filling limited region^[10]^, as defined in Equation:

$$\text{n}_{\text{t}}\text{ }\text{= }\frac{\text{2}\text{ε}_{\text{r}}\text{ε}_{\text{0}}\text{V}_{\text{TFL}}}{\text{q}\text{L}^{\text{2}}}$$

Where ε_0_ is the vacuum permittivity (8.854 × 10^−12^ F/m), ε_r_ is the relative dielectric constant of FAPbI_3_ (35), q is the electric charge with 1.6 × 10^−19^ C, and L is the thickness of the perovskite film. Linear least-squares fitting is performed for the three regions of the $\log\text{(J)-}\log\text{(V)}$ curve. The trap-filled limited voltage $\text{V}_{\text{TFL}}$ is precisely determined from the intersection of the two fitted straight lines, which is further substituted into the formula to calculate the trap state density $\text{n}_{\text{t}}$.

Figure S28. Steady-state output during measurements of the photocurrent density and PCE.

Figure S29. EIS curves of PSCs.

The equivalent circuit adopts a first-order RC model, in which the series resistance R_S_ is connected in parallel with the recombination branch. The recombination branch consists of the recombination resistance R_rec_ and chemical capacitance C_rec_ in parallel. The core expression of complex impedance is:

$\text{Z(ω) = }\text{R}_{\text{s}}\text{+}\frac{\text{R}_{\text{rec}}}{\text{1+jω}\text{R}_{\text{rec}}\text{C}_{\text{rec}}}$,

Where $\text{ω = 2πf}$ is the angular frequency, and f represents the test frequency. $\text{R}_{\text{s}}$ denotes the series resistance of the device, corresponding to the high-frequency intercept on the horizontal axis Z' in the Nyquist plot. $\text{R}_{\text{rec}}$ is the bulk recombination resistance of the device, equal to the diameter of the semicircle in the Nyquist plot. $\text{C}_{\text{rec}}$ stands for chemical capacitance, which is calculated from the characteristic relaxation frequency of the semicircle, with $\text{f}_{\text{0}}\text{ = }\frac{\text{1}}{\text{2π}\text{R}_{\text{rec}}\text{C}_{\text{rec}}}$.

The full frequency range from 1 Hz to 1 MHz was selected for testing, with priority given to fitting the complete semicircular region in the Nyquist plot. Based on the real part (Z') and imaginary part (Z'') of the complex impedance, the equivalent circuit model was fitted via the nonlinear least‑squares method to minimize the deviation between the fitted curve and experimental data. The fitted curve exhibits high consistency with the experimental data. The chi-square value ($\chi^{2}$) of the fitting is less than 10^-3^, indicating a high-precision fitting result.

Table S1．Photovoltaic parameters of n-i-p perovskite solar cells.

| Device Structure | *V*_OC_  (V) | FF  (%) | *J_SC_*  (mA cm^-2^) | PCE (%) | Ref. |
| --- | --- | --- | --- | --- | --- |
| ITO/SnO_2_/perovskite  /spiro-OMeTAD/Ag | 1.16 | 85.49 | 26.22 | 25.94 | ^[11]^ |
| ITO/PTAA/SAM  /perovskite/C_60_/BCP/Cu | 1.18 | 83.61 | 25.93 | 25.59 | ^[12]^ |
| FTO/TiO_2_/SnO_2_/perovskite  /spiro-OMeTAD/Ag | 1.18 | 83.93 | 26.0 | 25.82 | ^[13]^ |
| FTO/SnO_2_/perovskite  /spiro-OMeTAD/Au | 1.18 | 82 | 25.97 | 25.13 | ^[14]^ |
| ITO/T-SnO_2_/perovskite  /spiro-OMeTAD/MoO_3_/Ag | 1.18 | 82.74 | 25.81 | 25.09 | ^[15]^ |
| ITO/Al_2_O_3_/SnO_2_/perovskite/Spiro-OMeTAD/Au | 1.19 | 83.2 | 26.0 | 25.7 | ^[16]^ |
| ITO/ETL/MHP/HTL/Au | 1.17 | 85.71 | 26.17 | 26.13 | ^[17]^ |
| FTO/TiO_2_/perovskite  /spiro-OMeTAD/Au | 1.19 | 84.11 | 26.01 | 26.07 | ^[18]^ |
| FTO/SnO_2_/perovskite  /spiro-OMeTAD/Au | 1.17 | 85.42 | 26.39 | 26.40 | ^[19]^ |
| glass/FTO/SnO2/perovskite/spiro-OMeTAD/Au | 1.182 | 84.0 | 26.3 | 26.2 | ^[20]^ |
| FTO/SnO_2_/perovskite  /spiro-OMeTAD/Au | 1.179 | 85.82 | 26.30 | 26.61 | ^[21]^ |
| glass/FTO/continuously graded SnO2/FACsPbI3 perovskite/Spiro-OMeTAD/Au | 1.215 | 86.13 | 26.41 | 27.64 | ^[22]^ |
| FTO/SnO_2_/perovskite/spiro-OMeTAD/Au | 1.196 | 83.96 | 26.20 | 26.31 | ^[23]^ |
| FTO/WE-SnO_2_/  perovskite/spiro-OMeTAD/Au | 1.21 | 85 | 25.67 | 26.4 | ^[24]^ |
| FTO/SnO2/FAPbI3/4-MeO-PEAI/Spiro-OMeTAD/Au | 1.196 | 82.95 | 26.30 | 26.13 | ^[25]^ |
| NP-FTO/ALD-SnO₂/SC-SnO₂/perovskite/spiro-OMeTAD/Au | 1.203 | 84.49 | 25.98 | 26.40 | ^[26]^ |
| FTO glass/c-TiOx/m-TiOx/ FAPbI3/spiro-OMeTAD/Au | 1.176 | 84.478 | 26.391 | 26.2 | ^[27]^ |
| FTO/TiO_2_/perovskite  /spiro-OMeTAD/Au | 1.19 | 83.66 | 26.87 | 26.67 | This work |

Table S2．EIS fitting parameters of the PSCs.

| Sample | *R_S_* (Ω) | *C_tr_* (F) | *R_rec_* (Ω) |
| --- | --- | --- | --- |
| Control | 8.91 | 1.79×10^−7^ | 74.65 |
| NaCl | 8.57 | 1.75×10^−7^ | 135.7 |
| C_10_H_21_NaO_3_S | 8.08 | 1.24×10^−7^ | 457.3 |

**References**

[1]	S. J. Clark, M. D. Segall, C. J. Pickard, P. J. Hasnip, M. I. J. Probert, K. Refson, M. C. Payne, *First principles methods using CASTEP*, *Z. Kristallogr. Cryst. Mater.* **2005**, *220*, 567-570, https://doi.org/10.1524/zkri.220.5.567.65075.

[2]	J. P. Perdew, K. Burke, M. Ernzerhof, *Generalized Gradient Approximation Made Simple*, *Phys. Rev. Lett.* **1996**, *77*, 3865-3868, https://doi.org/10.1103/PhysRevLett.77.3865.

[3]	P. J. Hasnip, C. J. Pickard, *Electronic energy minimisation with ultrasoft pseudopotentials*, *Comput. Phys. Commun.* **2006**, *174*, 24-29, https://doi.org/10.1016/j.cpc.2005.07.011.

[4]	J. P. Perdew, J. A. Chevary, S. H. Vosko, K. A. Jackson, M. R. Pederson, D. J. Singh, C. Fiolhais, *Atoms, molecules, solids, and surfaces: Applications of the generalized gradient approximation for exchange and correlation*, *Phys. Rev. B* **1992**, *46*, 6671-6687, https://doi.org/10.1103/PhysRevB.46.6671.

[5]	J. D. Head, M. C. Zerner, *A Broyden—Fletcher—Goldfarb—Shanno optimization procedure for molecular geometries*, *Chem. Phys. Lett.* **1985**, *122*, 264-270, https://doi.org/10.1016/0009-2614(85)80574-1.

[6]	Z. Tang, T. Bessho, F. Awai, T. Kinoshita, M. M. Maitani, R. Jono, T. N. Murakami, H. Wang, T. Kubo, S. Uchida, H. Segawa, *Hysteresis-free perovskite solar cells made of potassium-doped organometal halide perovskite*, *Sci. Rep.* **2017**, *7*, 12183, https://doi.org/10.1038/s41598-017-12436-x.

[7]	J. K. Nam, S. U. Chai, W. Cha, Y. J. Choi, W. Kim, M. S. Jung, J. Kwon, D. Kim, J. H. Park, *Potassium Incorporation for Enhanced Performance and Stability of Fully Inorganic Cesium Lead Halide Perovskite Solar Cells*, *Nano Lett.* **2017**, *17*, 2028-2033, https://doi.org/10.1021/acs.nanolett.7b00050.

[8]	Y. B. Cao, Y. Fu, Y. Zhou, X. Qiu, D. Zhang, Y. Ding, Y. Xie, B. Ren, Q. Shan, P. F. Chan, W. Tang, F. Xue, X. Sun, K. Zhou, J.-F. Liao, Z. Jin, Q. Zhang, J. Wang, D.-B. Kuang, X. Lu, Y. Lin, H. Zeng, Z. Fan, *Ultrapure and efficient electroluminescence in alkali metal doped inorganic perovskite quantum wires arrays*, *Nat. Commun.* **2025**, *16*, 6053, https://doi.org/10.1038/s41467-025-61085-6.

[9]	D.-Y. Son, S.-G. Kim, J.-Y. Seo, S.-H. Lee, H. Shin, D. Lee, N.-G. Park, *Universal Approach toward Hysteresis-Free Perovskite Solar Cell via Defect Engineering*, *J. Am. Chem. Soc.* **2018**, *140*, 1358-1364, https://doi.org/10.1021/jacs.7b10430.

[10]	W.-M. Gu, K.-J. Jiang, X. Jiao, C.-Y. Gao, X.-H. Fan, L.-M. Yang, Y. Song, *In-Situ Cyclized Polyacrylonitrile as an Electron Selective Layer for n-i-p Perovskite Solar Cell with Enhanced Efficiency and Stability*, *Angew. Chem. Int. Ed.* **2024**, *63*, e202403264, https://doi.org/10.1002/anie.202403264.

[11]	L. Li, J. Xu, L. Fang, Z. Feng, H. Huang, Y. Wang, Y. Guo, S. Kang, H. Wang, Y. Han, Y. Ji, H. Zhang, Y. Ding, M. K. Nazeeruddin, B. Ding, X. Zhang, J. Peng, *Active Passivation Charge Transport in n-i-p Perovskite Solar Cells Approaching 26% Efficiency*, *Adv. Mater.* **2025**, *37*, 2503903, https://doi.org/10.1002/adma.202503903.

[12]	Z. Zhang, J. Yang, Z. Wu, X. Liu, H. Zhong, X. Wang, J. Su, H. Wang, J. Lee, X. Li, H. Lin, *Revealing Bifunctional Molecules Induced Adequate DMSO Extraction in Sequential Crystallization toward Efficient and Robust Flexible Perovskite Solar Cells*, *Adv. Funct. Mater.* **2026**, *36*, e22596, https://doi.org/10.1002/adfm.202522596.

[13]	Y. Wang, C. Deng, J. Wu, W. Pan, M. Guo, J. Xu, Y. Du, F. Yu, Z. Lan, *Synergistic Regulation of SnO_2_/Perovskite Buried Interface by Pyridine Derivative Molecular Bridging for Photovoltaic Performance Optimization*, *Adv. Funct. Mater.* **2026**, e75104, https://doi.org/10.1002/adfm.75104.

[14]	H. W. Chun, S. Y. Lee, S. E. Yoon, G. G. Jeon, V. M. Arivunithi, S. J. Shin, M. J. Choi, B. Kim, M.-H. Lee, D. Jeon, T. Kim, J.-Y. Lee, J. H. Kim, *Fluorine-Doping of Mesoporous TiO_2_ Enables Efficient Defect Passivation for High-Efficiency and Stable Perovskite Solar Cells*, *Adv. Sci.* **2026**, e24360, https://doi.org/10.1002/advs.202524360.

[15]	J. Cao, Q. Chen, W. Wu, J. Fu, Z. Zhang, L. Chen, R. Wang, W. Yu, L. Wang, X. Nie, J. Zhang, Y. Zhou, B. Song, Y. Li, *Chlorinated-Ti_3_C_2_TF as a dual-functional buried interface on SnO_2_ electron-transporting layers for 25.09% high-performance n–i–p perovskite solar cells*, *Energy Environ. Sci.* **2024**, *17*, 3454-3469, https://doi.org/10.1039/D4EE00196F.

[16]	D. Zhang, B. Li, P. Hang, J. Xie, Y. Yao, C. Kan, X. Yu, Y. Zhang, D. Yang, *Mitigated front contact energy barrier for efficient and stable perovskite solar cells*, *Energy Environ. Sci.* **2024**, *17*, 3848-3854, https://doi.org/10.1039/D4EE00316K.

[17]	L. Zhang, Q. Dong, Z. Ying, P. Xu, S. Rong, S. Jin, Y. Shi, W. Tian, *KOH‑Modified SnO_2_ Buried Interfacial Crystallization Engineering for Efficient Flexible Perovskite Solar Cells*, *Adv. Funct. Mater.* **2026**, e75347, https://doi.org/10.1002/adfm.75347.

[18]	Y. Wang, L. Zhang, K. Li, Y. Wang, Y. Tao, X. Lu, S. Tsang, H.-C. Kuo, J. You, A. K. Y. Jen, S. F. Liu, *A Bimolecular Co-Anchoring Strategy for Constructing Hydrogen Networks to Stabilize Perovskite Solar Cells*, *Adv. Energy Mater.* **2026**, *16*, e04844, https://doi.org/10.1002/aenm.202504844.

[19]	M. Li, B. Jiao, Y. Peng, J. Zhou, L. Tan, N. Ren, Y. Ye, Y. Liu, Y. Yang, Y. Chen, L. Ding, C. Yi, *High-Efficiency Perovskite Solar Cells with Improved Interfacial Charge Extraction by Bridging Molecules*, *Adv. Mater.* **2024**, *36*, 2406532, https://doi.org/10.1002/adma.202406532.

[20]	Kim, K., Yang, S., Kim, C., Park, J., Jeong, S., Kim, Y., ... & Min, H. Non-volatile solid-state 4-(N-carbazolyl) pyridine additive for perovskite solar cells with improved thermal and operational stability. Nat Energy 10, 1427–1438 (2025). https://doi.org/10.1038/s41560-025-01864-z;

[21]	Tang, X., Yang, C., Xu, Y., Xia, J., Li, B., Li, M., ... & Zheng, X. Enhancing the efficiency and stability of perovskite solar cells via a polymer heterointerface bridge. Nat. Photon. 19, 701–708 (2025). https://doi.org/10.1038/s41566-025-01676-3;

[22]	Wang, D., Li, S., Ding, Z., Xu, J., Chen, X., Zheng, Q., ... & Yuan, M. Continuously graded-doped SnO2 for efficient n–i–p perovskite solar cells. Nature 654, 69–75 (2026). https://doi.org/10.1038/s41586-026-10587-4;

[23]	Gao, Y., Liu, H., Song, Z., Chen, Y., Yang, L., Hu, Z., ... & Liu, Y. Spontaneous 2D perovskite formation at the buried interface of perovskite solar cells enhances crystallization uniformity and defect passivation. Nat. Photon. 20, 178–185 (2026). https://doi.org/10.1038/s41566-025-01797-9;

[24]	Seo, G., Yoo, J. J., Nam, S., Lee, D. S., Gao, S., Kim, B. K., ... & Shin, S. S. Efficient and luminescent perovskite solar cells using defect-suppressed SnO2 via excess ligand strategy. Nat Energy 10, 774–784 (2025). https://doi.org/10.1038/s41560-025-01781-1;

[25]	Zhang, K., Wang, Y., Guo, L., Tao, M., Guo, H., Zhang, X., ... & Song, Y. Surface diffusion engineering of a 2D perovskite layer for efficient perovskite photovoltaics. Nat. Synth 4, 1386–1395 (2025). https://doi.org/10.1038/s44160-025-00865-w;

[26]	Ge, Y., Zheng, L., Wang, H., Gao, J., Yao, F., Wang, C., ... & Fang, G. Suppressing wide-angle light loss and non-radiative recombination for efficient perovskite solar cells. Nat. Photon. 19, 170–177 (2025). https://doi.org/10.1038/s41566-024-01570-4;

[27]	Li, Q., Liu, H., Hou, C. H., Yan, H., Li, S., Chen, P., ... & Zhu, R. Harmonizing the bilateral bond strength of the interfacial molecule in perovskite solar cells. Nat Energy 9, 1506–1516 (2024). https://doi.org/10.1038/s41560-024-01642-3.
